# HIV-1 Tat – TLR4/MD2 interaction drives the expression of IDO-1 in monocytes derived dendritic cells through NF-κB dependent pathway

**DOI:** 10.1038/s41598-020-64847-y

**Published:** 2020-05-18

**Authors:** Elmostafa Bahraoui, Manutea Serrero, Rémi Planès

**Affiliations:** 1 INSERM, U1043, CPTP, CHU purpan, Toulouse, France; 2 CNRS, U5282 CPTP, CHU purpan, Toulouse, France; 3 Université Paul Sabatier, CPTP, CHU purpan, Toulouse, France

**Keywords:** Gene regulation in immune cells, Signal transduction

## Abstract

In the present study we showed that HIV-1 Tat protein stimulated the expression of Indoleamine 2,3 dioxygenase (IDO) -1 in human monocytes derived dendritic cells (MoDC) but not IDO-2 by acting directly at the cell membrane level. This induction of IDO-1 is dependent on the secondary structure of Tat protein, since stimulation with a chemically oxidized Tat protein loses its capacity to induce the production of IDO-1. Among the variety of candidate receptors described for Tat, we demonstrated that Tat protein interacted physically with TLR4/MD2 complex. Strikingly, blockade of Tat-TLR4 interaction by anti-TLR4 antibodies (clone HTA125), LPS-RS, a known TLR4 antagonist, or by soluble recombinant TLR4/MD2 complex inhibited strongly or totally the capacity of Tat to induce IDO-1 in MoDC while such treatments had no effect on IFN-γ-induced IDO-1. Furthermore, we showed that the activation of the transcription factor NF-κB by Tat is essential for the production of IDO-1 by human MoDC. Indeed, Tat activated NF-κB pathway in MoDC as demonstrated by the phosphorylation of p65 in Tat-treated MoDC. Further, we demonstrate that the stimulation of IDO-1 by Tat or by IFN-γ was totally or partially inhibited in the presence of NF-κB inhibitor respectively. These results suggest that Tat and IFN-γ act probably by two distinct mechanisms to induce the production of IDO-1. Our results clearly demonstrated that, although TLR4 pathway is necessary for Tat-induced IDO-1 in MoDC, it seems not to be sufficient since stable transfection of a functional TLR4/MD2 pathway in HEK or HeLa cell lines which are endogenously defectives for TLR4, did not restore the capacity of Tat to induce IDO-1 while IFN-γ treatment induces IDO-1 in HeLa cells independently of TLR4 pathway. These results suggest the involvement of additional stimuli in addition to TLR4 pathway which remain to be identified. Altogether our results demonstrated that, in human MoDC, HIV-1 Tat protein induced IDO-1 expression and activity in a NF-κB dependent-manner by recruiting TLR4 pathway.

## Introduction

HIV-1 Tat protein, one of the six accessory proteins encoded by the viral genome is considered with Rev protein as the two essential viralregulatory factors for productive viral replication *in vitro*. The Tat gene product is a polypeptide of 14 kDa, consisting of 86 to 104 amino-acids structured in six structural and functional identified domains^1,2^. Tat protein plays a crucial role in the transactivation of HIV-1 long terminal repeat (LTR) promoter, a key step essential for the expression of structural and accessory viral genes and transcription of HIV-1 genomic RNA. This transactivation is mediated by the binding of Tat protein on the Tat-associated region (TAR) of nascent viral mRNA^2,3^. However, in addition to the essential role of Tat protein in the productive viral cycle, HIV-1 Tat protein is also considered as an important pathogenic factor by acting on different cell types beyond their sensitivity and permissivity to HIV-1. Indeed, several pathogenic effects were attributed to Tat protein, including neurotoxicity at the central nervous system^4–6^, apoptosis of CD4 positive cells^7,8^, pharmacological dysfunctions of gastrointestinal tract (GALT) from HIV-1 infected patients and a large spectra of immunosuppressive effects which seems to play an important role in the weakening of the immune system since asymptomatic phase^2,9,10^. Several of these immunosuppressive effects are mediated by the action of Tat-induced cytokines/chemokines and immunosuppressive factors^2,10^. One of the important singularity that characterize HIV-1 Tat protein, is the fact that despite the absence of signal sequence, this protein is secreted by HIV-1 infected cells and can be found at nanomolar concentrations in the sera of HIV-1 infected patients^11–13^.

Many studies, including ours, have shown that Tat protein induces IL-10, a highly immunosuppressive cytokine, in human monocytes, macrophages and dendritic cells^14–29^. We have shown that the induction of IL-10 in human monocytes is mediated by an action of Tat protein at the plasma membrane level without requiring its intracellular internalization^28^. To evaluate the involved cell surface receptor, we explored the implication of the potential receptors reported for Tat, including CD26 receptor^30^ L-Type calcium channel^31^, the integrin αvβ3 and α5β1 of dendritic cells^32^, membrane lipids or Flk-1/KDR receptor^33^. However, a neutralizing antibody based screen revealed that none of these potential Tat receptors seems to be involved. Thus, we hypothesized the involvement of TLR4 as a potential receptor due to the fact that monocytes are TLR4 positive, produce IL-10 via TLR4 pathway and Tat-induced IL-10 was totally inhibited in the presence of HTA125 monoclonal blocking anti-TLR4 antibodies^14^. In addition to IL-10, Tat protein induced the up regulation of PD-L1, an immunosuppressive factor, in human monocytes derived dendritic cells. The immunosuppressive action of PD-L1 is mediated following its interaction with PD-1 which lead to the inhibition of TCR signaling^34^. The mechanism of Tat-induced up regulation of PD-L1 is independent of a direct Tat-cell contact as demonstrated by the capacity of Tat-conditioned medium to induce PD-L1 up regulation^35^, but is mediated by an indirect action involving Tat-induced TNF-α^35^.

In addition to the two immunosuppressive factors, IL-10 and PD-L1, Tat protein also induced in MoDC the production of indoleamine, 2,3 dioxygenase-1 (IDO-1)^36–38^. Indoleamine 2,3 dioxygenase activity is carried by 3 separate genes. The products of these genes are intracellular with monomeric structures of 43 kDa for IDO-1 and IDO-2 or homotetrameric of 134 kDa for TDO. These proteins are heme enzymes responsible for the degradation of tryptophan to N-formyl-kynurenine. This first step in the catabolism of L-tryptophan represents the rate limiting step in the kynurenine pathway, since all the other enzymes involved in this pathway are constitutively produced^39^. Therefore, the gene product expression of IDO-1, IDO-2 and TDO must be, in general, induced. IDO-1 is rather induced by anti-inflammatory cytokines TGF-β^39,40^ or pro-inflammatory stimuli such as IFN-γ and potentiated in the presence of TNF-α, IL-1β, and IL-2. IDO-1 can be also induced by activated T-cells signals^41–43^ and plays a key role in the modulation of the physiological concentrations of L-tryptophan under inflammatory conditions. At a functional level, we previously showed that Tat-induced IDO-1 expression is associated with a potent immunosuppressive phenotype *in-vitro* characterized by a loss of the capacity of MoDC to activate the proliferation of autologous T cells in a T cells assay^36^.

Because, Tat-induced IDO-1 is dependent on a direct contact between Tat and MoDC and cannot be restored by stimulation with Tat-conditioned medium, the aim of the present study was to explore the involvement of TLR4, as potential receptor candidate, in the induction of IDO-1 in human monocyte-derived dendritic cells, following the treatment by HIV-1 Tat protein.

## Materials and Methods

### Ethics, method and competing interest statements

The use of human cells was approved by the Research Ethical Committee, Haute-Garonne, France. Buffy coats were provided anonymously by the EFS (établissement français du sang, Toulouse, France). Written informed consent was obtained from each donor under EFS contract N° 21/PVNT/TOU/INSERM01/2011-0059, according, to “Decret N° 2007-1220 (articles L1243-4, R1243-61)”. All the authors concur with the submission and have no competing interests. We confirm that all methods were performed in accordance with the relevant guidelines and regulation. All the authors concur with the submission and declare no competing interest. The manuscript, which has not been submitted elsewhere, contains human studies which conform to the Guides for IRB and IACUC published by the US National Institute of Health.

### Generation of monocyte-derived DCs

Peripheral Blood Mononuclear Cells (PBMCs) were isolated from buffy coat of healthy donors obtained from the EFS Toulouse Purpan, France as described previously^36^. Briefly, PBMCs were isolated by centrifugation using standard Ficoll-Paque density (GE Healthcare). The blood was diluted 1:1 in phosphate-buffered saline (PBS) pre-warmed to 37 °C and carefully layered over the Ficoll-Paque gradient. The tubes were centrifuged for 25 min at 2000 rpm, at 20 °C. The cell interface layer was harvested carefully, and the cells were washed twice in PBS (for 10 min at 1200 rpm followed by 10 min at 800 rpm) and re-suspended in MACS buffer (PBS pH 7.4, 0.5% BSA, 2mM DTA). Monocytes were separated from lymphocytes by positive selection using CD14 + isolation kit (Myltenyi biotec). To allow differentiation into monocyte-derived DCs (MoDC), cells were cultured in RPMI medium (GIBCO) supplemented with 10% FCS (Invitrogen), 100 IU/ml penicillin, 100 µg/ml streptomycin, 10 ng/ml GM-CSF and 10 ng/ml IL-4. After 5 days of culture loosely adherent cells were collected by gentle pipetting and used as immature DCs in our experiments. Over 90% of cells had the standard phenotype of immature DCs: CD1a+, CD14−, CD80+, CD86+, CD83−, HLA-DR+.

### HeLa and HEK cell line expressing TLR4/MD2-CD14

HeLa and HEK cell lines stably expressing human TLR4, MD2 and CD14 or HEK and HeLa null control were purchased from Imgenex, Novus Biologicals and Invivogen respectively, and maintained in culture according to the manufacturer’s instructions.

### Recombinant and synthetic Tat proteins

Recombinant Tat protein from HIV-1 group M subtype B (isolate SF2) (www.uniprot.org/uniprot/P04614) full length protein (Tat 1-101), core domain (Tat 30-72) or N-terminal Tat fragments (Tat 1-45) fused to glutathione S-transferase (GST) were produced and purified in our laboratory as previously described^28^.

Chemically synthesized Tat (aa 1–86) sequence from HIV-1 group M subtype B (isolate LAI) (www.uniprot.org/uniprot/P04610) and its oxidized counterpart were produced as described previously^3^. The level of endotoxin in all recombinant GST-Tat proteins was tested using the Limulus Amebocyte Lysate assay (Bio-Sepra, Villeneuve la Garenne, France) and was shown to contain less than 0.3 EU/µg, the limit of detection of this assay.

### Antibodies, chemicals and cytokines

Chemical products: Lipopolysaccharide (LPS), from *E. coli* serotype R515, was purchased from Alexis biochemicals. LPS-RS, from *Rhodobacter sphaeroides* and CLI-095 were purchased from Invivogen. The NF-κB inhibitor Bay 11-7082 was purchased from Calbiochem. Recombinant human TLR4/MD2 and CD14 proteins were purchased from R&D system. L-tryptophan, L-Kynurenine, and Ehrlich’s reagent were from Sigma-Aldrich.

Recombinant cytokines and antibodies: Recombinant human cytokines IFN-γ were purchased from eBioscience. Recombinant human granulocyte macrophage-colony-stimulating factor (GM-CSF) and interleukin-4 (IL-4) were purchased from HumanZyme or Miltenyi Biotech.

For blocking experiments Mouse monoclonal anti-TLR4/MD2 (HTA 125), anti-TLR2 (TL2.1) and mouse IgG isotype control purified antibodies were purchased from eBioscience.

For cell surface labelling fluorochromes conjugated antibodies anti-CD1a-FITC, anti-CD14-PE, anti-CD80-FITC, anti-CD83-FITC, anti-CD86-PE, anti-HLA-DR-FITC and isotype control were purchased from Biolegend. Anti-TLR4-PE, anti-PD-L1-APC and isotype control were purchased from eBioscience.

For Tat-TLR4 binding assay Anti-Tat antibodies were obtained from ANRS. Anti-GST antibodies were produced in our laboratory as described by^58^. Anti-TLR4 antibodies were purchased from eBioscience. HRP conjugated secondary antibodies were purchased from Dako. Alexa Fluor-555 and Alexa Fluor-488 were purchased from Invitrogen.

For immunoblot experiments mouse monoclonal anti-human-IDO-1 antibodies, rabbit monoclonal anti-human-IDO-1 antibodies and rabbit monoclonal anti-human-IDO-2 antibodies were purchased from Abcam. Monoclonal mouse anti-β-actine antibodies (clone AC-15) were purchased from Sigma-Aldrich. Anti-GAPDH (GTX100118) were purchased from GeneTex. Anti-Phospho-NF-κB p65 (Ser536) (clone 93H1) and anti-p65 (clone D14E12) were purchased from cell signaling Technology. Secondary antibodies coupled with HRP were from Dako.

### Treatment of monocyte-derived dendritic cells with Tat

After differentiation, MoDCs were resuspended in RPMI complete medium supplemented with 10% FCS containing penicillin (100 IU/ml) and streptomycin (100 µg/ml) at density of 1.10^6^ cells/ml and seeded in 6-well plates at 2.10^6^ cells/wells. Cells were then treated as indicated with either Tat protein, LPS, IFN-γ or otherwise specified for a period of 24 h. Cells were then collected and centrifuged for 10 min at 1200 rpm, cell culture supernatants were kept frozen until cytokine quantification, while cells were used for the quantification of IDO expression and activity. For TLR4 blockade experiments, MoDCs were pre-treated with either anti-TLR4-MD2, LPS-RS, or incubated with recombinant TLR4-MD2 (5 µg/ml) for 1 h before stimulation with Tat, IFN-γ or other stimuli as indicated. For NF-κB inhibition experiments, MoDCs were incubated with Bay 11-7082 for 30 min before stimulation.

### Analysis of IDO expression and activity

IDO protein expression in MoDCs was investigated by immunoblot and IDO activity was quantified by Ehrlich’s Assay as described previously^36^. To evaluate the activity of IDO in catabolizing tryptophan into kynurenine, MoDCs were resuspended in Hanks buffered saline solution (HBSS) supplemented with 500 µM L-tryptophan and incubated for 2 to 4 hours at 37 °C. Supernatants were then harvested and kynurenine was quantified by Ehrlich’s Assay. Briefly, supernatant was cleared of its protein contents by treatment with 30% trichloroacetic acid followed by 5 min of centrifugation at 10000 rpm. Then, 100 µl of soluble phase was mixed with 100 µl of Ehrlich’s reagent (25 mg/ml of 4-dimethylaminobenzaldehyde in glacial acetic acid) in 96-well plates. The OD at 492 nm was measured and kynurenine concentrations were calculated using a kynurenine standard curve. IDO protein expression was assessed by immunoblot as described previously^36^. Briefly MoDCs were lysed in lysis buffer (20 mM Tris-HCl, 150 mM NaCl, 1 mM EDTA, 0.5% NP-40, 0.2% SDS, pH 7.4 supplemented with a protease inhibitor cocktail). Proteins were then separated by 12% SDS-PAGE and transferred to nitrocellulose membranes. After saturation in Tris-buffered saline (TBS, pH8) containing 0.05% Tween 20 and 5% non-fat milk for 1 hour at room temperature, membranes were further incubated with anti-human IDO-1 antibodies (3 µg/ml) at 4 °C overnight and secondary antibody for 1 h at room temperature. Membranes were washed 3 times with TBS containing 0.1% Tween 20, between each step. Finally, immunoreactive bands were labelled using a chemiluminescent substrate (Pierce). When needed, membranes were striped by a treatment of 20 minutes in glycine 0.1 M, 0.1% NP40, 1% SDS, pH 2.2 buffer before their use for β-actin detection.

### Tat-TLR4 interaction assays

Molecular assay: Tat-TLR4 binding was investigated following a solid-phase binding assay. Recombinant TLR4/MD2 complexes (1 µg/ml) were coated on 96 maxisorp well plates overnight at 4 °C. Plates were saturated for 1 hour at room temperature using diluent assay solution from eBioscience. Then, plates were incubated sequentially with increasing concentrations (0.1-1 µM) of GST or GST-Tat protein (for 2 hours at room temperature), anti-GST primary antibody (1 hour at room temperature) and anti-primary antibody conjugated to peroxidase (1 hour at room temperature). 3 washes using PBS containing 0.05% Tween 20 (wash buffer) were performed between each step. Finally, plates were incubated with the enzyme substrate (TMB) and the reaction was stopped by H_2_SO_4_ (4 N). Absorbance was read at 450 nm with a wavelength correction at 570 nm.

Cell binding assay: HEK cells not transfected (HEK Null) or stably transfected with CD14, MD2 and TLR4, (HEK-CD14-MD2-TLR4) were cultured on glass coverslides 12-mm round diameter. At 60–80% confluence, the cells were treated with GST-Tat1-101 or GST alone as a control for 60 min at 4 °C. After 3 washes with PBS, cells were fixed during 15 min with paraformaldehyde at 4%. Then, in order to inhibit autofluorescence, free aldheydes were saturated by NH_4_Cl (50 mM) as quenching agent. After further washing, cells were incubated for 30 min with PBS buffer containing bovine serum albumin at 5%. Then, cells were stained with anti-GST and anti-TLR4 antibodies or the corresponding isotypic control antibodies. After 3 washes with PBS, secondary antibodies conjugated with Alexa Fluor-555 and Alexa Fluor-488 were used to detect anti-GST and anti-TLR4 antibodies respectively. Cells nuclei were stained with DAPI. Cells were imaged using confocal Zeiss LSM 710 (Image Core facility, CPTP, Toulouse) using a 63x objective. Colocalization and subcellular localization were analyzed and processed with ImageJ software. Colocalization images were measured and quantified with JacoP software.

### NF-κB immunoblot

After 1 h of treatment cells were collected, centrifuged for 10 min at 1200 rpm, cell pellet was resuspended in phosphate-protective lysis buffer (20 mM Tris-HCl pH 7.4, 150 mM NaCl, 1.5 mM MgCl_2_, 10% Glycerol, 0.5 mM EDTA, KCl 10 mM, 0.5% Triton X100, 10 mM β-mecropto-ethanol, and containing 1X of protease inhibitor cocktail (Roche) and phosphostop (Roche)). Cells were lysed by pipetting up and down several times and then incubated for 20 min on ice. Cell lysate were separated by SDS-PAGE as described previously^36^. Immunoblot were performed by using anti-Phospho-NF-κB p65 (Ser536), anti-p65 total and anti-GAPDH as loading control.

### Cytokine quantifications by ELISA

Quantification of IL-10 cytokine in MoDC supernatant and quantification of human CXCL8 chemokine in HEK and HeLa cell’s supernatants were performed by ELISA as described previously^36,44^.

### Statistical analyses

Statistical analysis was performed using GraphPad Prism software v.5. All results are expressed as means +/− SD. All experiments were performed a minimum of three times. Differences in the means for the different groups were tested using one-way ANOVA followed by Bonferroni post hoc test. A p-value <0.05 was considered statistically significant. Statistical significance comparing different groups is denoted with * for p < 0.05, **p < 0.01, ***p < 0.001, ns non-significant.

## Results

### HIV-1 Tat protein induces the production of IDO-1 in human MoDC

**Figure 1:**
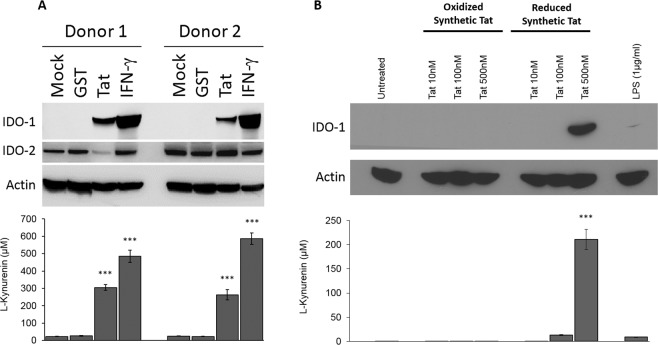
HIV-1 Tat protein induces the expression and activation of IDO-1 in MoDC in specific manner. (**A**) MoDC from two different healthy donor (Donor 1 and Donor 2) were incubated with GST protein (100 nM), HIV-1 Tat protein (100 nM), IFN-γ (1000 u/ml), or treated with PBS (Mock). 24 hrs after treatment cells were collected, lysed and analysed by SDS-PAGE and Western-Blot for IDO-1, IDO-2 and actin protein expression using specific antibodies. IDO activity (kynurenine production) was measured by Ehrlich’s assay following a 2 h incubation in HBSS completed with L-tryptophane (500 µM). (**B**) MoDC were treated with escalating among of synthetic HIV-1 Tat protein in either oxidized or reduced form (10-500 nM). Untreated cells and LPS (1 µg/ml) treated cells were used as control. After 24 h of treatment MoDC were collected and analyzed for IDO-1 protein expression by immunoblot and IDO activity. The data represent means and SD for three independent experiments. Statistical significance was analyzed by one-way ANOVA follow by Bonferroni post tests and is marked as follows: *P < 0.05; **P < 0.01; ***P < 0.001; ns, non significant. All bars were compared to those for untreated cells.

In agreement with our previous study^36^, we showed that HIV-1 Tat protein, used as recombinant GST fusion protein induced the production of IDO-1 but not IDO-2 in human monocyte derived dendritic cells (MoDC) (Fig. 1A). This Tat-induced IDO-1 was enzymatically active, as shown by its capacity to oxidize, in a colorimetric *in vitro* assay, the L-Tryptophan amino acid into L-kynurenine^36^ (Fig. 1A lower panel). As negative controls, we showed that no significant band related to IDO proteins expression nor IDO enzymatic activity were detected from untreated or GST-treated MoDC (Fig. 1A upper and lower panels). To better characterize the specificity and the structure-activity relationships of the effect obtained with the recombinant Tat protein, we used a chemically synthetic Tat protein in either a reduced or oxidized form. Results depicted in Fig. 1B showed that only reduced form of Tat protein, which mimics the native like protein, is able to induce the expression of IDO-1 as shown both by the detection of the protein by immunoblot assay and by its capacity the metabolite L-Tryptophan leading to the production of L-kynurenin (Fig. 1B, upper and lower panels). These results indicate that the activity of Tat protein to induce IDO-1 expression seems to be dependent, at least, on its secondary structure. In fact, the oxidation of the SH groups, blocks the formation of the disulfide bridges which are necessary for the normal folding of the Tat protein in order to adopt the native-like structure. Furthermore, the activity found with the synthetic Tat protein, which has never been in the presence with bacterial endotoxins, is a valuable argument of the intrinsic activity of the Tat protein in addition to the fact that the recombinant GST-Tat protein was also tested as endotoxin free, less than 0.3 EU/µg, the limit of the detection assay used. Note also that the treatment of MoDC with LPS has only a weak effect on the expression of IDO-1 (Fig. 1B, upper and lower panels).

Altogether, our results clearly showed that HIV-1 Tat protein induced active IDO-1 expression in human MoDC and demonstrated that this activation is dependent at least on the secondary conformation of HIV-1 Tat protein.

### Tat -TLR4 interaction is essential for IDO-1 induction

**Figure 2:**
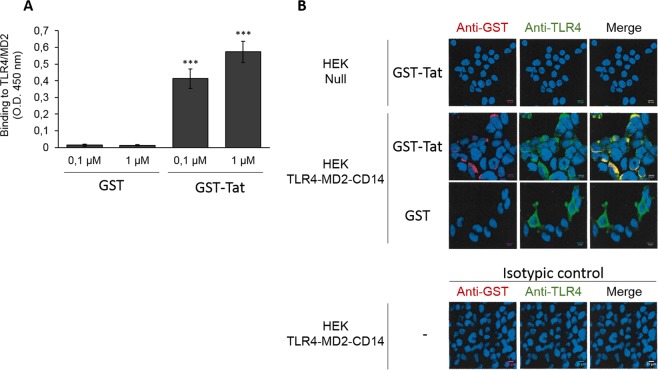
Tat specifically bind to TLR4/MD2. (**A**) Characterization of GST-Tat binding to recombinant TLR4/MD2 (Coating 1 µg/ml) by ELISA. GST was used as control. The data represent means and SD for three independent experiments. Statistical significance was analyzed by one-way ANOVA follow by Bonferroni post tests and is marked as follows: *P < 0.05; **P < 0.01; ***P < 0.001; ns, non significant. (**B**) Cell surface binding assay: HEK (Null) or HEK TLR4-CD14-MD2 were incubated with GST-Tat1-101 or GST alone for 60 min at 4 °C. Cells were stained with anti-GST and anti-TLR4 antibodies or the corresponding isotypic control antibodies. Secondary antibodies conjugated with Alexa Fluor-555 and Alexa Fluor-488 were used to detect anti-GST and anti-TLR4 antibodies respectively. Cells nuclei were stained with DAPI. Cells were imaged using confocal Zeiss LSM 710 (Image Core facility, CPTP, Toulouse) using a 63x objective. Colocalization and subcellular localization were analyzed and processed with ImageJ software.

We have demonstrated that a direct contact between Tat and MoDC is essential for IDO-1 induction^36^. So we then asked the question of the nature of the involved cell membrane receptor. Taking into account that Tat protein is also able to induce the production of IL-10 and PD-L1, two immunosuppressive factors, by activating TLR4 dependent pathways, we advanced the hypothesis of the implication of TLR4/MD2 as a potential Tat receptor responsible for the induction of IDO-1 expression. To this end we (i) firstly, validated and confirmed the capacity of Tat to interact with TLR4 in a solid phase binding assay in which recombinant TLR4/MD2 complexes (1 µg/ml) were incubated with escalating concentrations (0.1–1 µM) of total GST-Tat protein while no significant binding was observed with the GST (Fig. 2A). (ii) Secondly, we demonstrated that Tat protein is also able to interact with TLR4 in a cell-based assays. To this end, we showed that Tat protein interacts at cell membrane level with HEK cell lines stably transfected with TLR4-MD2-CD14, but not with non transfected cells (HEK null). As negative control, we showed that no cell surface binding was observed with GST protein alone (Fig. 2B).

**Figure 3:**
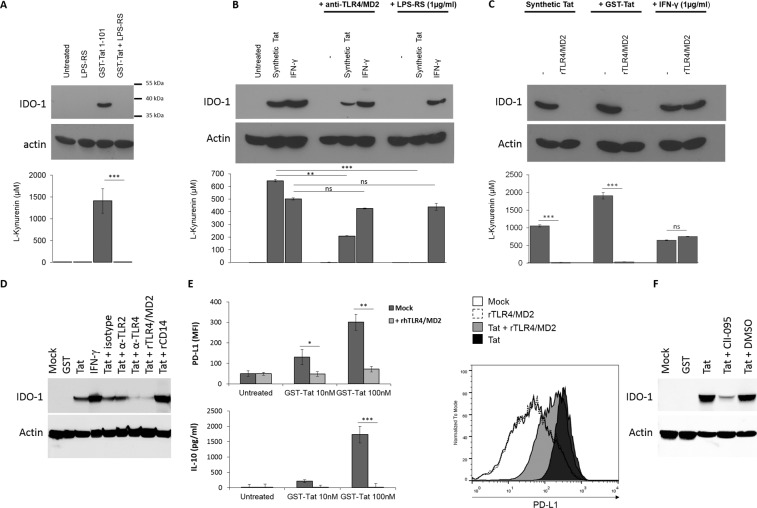
HIV-1 Tat protein induces the expression of IDO-1 in a TLR4/MD2 dependent mechanism. (**A**) MoDC were incubated or not with recombinant GST-Tat 1-101 (100 nM) in the presence or absence of the TLR4 antagonist LPS-RS (1 µg/ml). Untreated cells and LPS-RS treated cells where included as control (**B**) MoDC were incubated or not with anti-TLR4/MD2 (HTA125) 10 µg/ml or with the TLR4 antagonist LPS-RS (1 µg/ml) or (**C**) 5 µg/m of recombinant TLR4/MD2. After 1 h, cells were treated with synthetic HIV-1 Tat 1-86 protein reduced form (500 nM), recombinant GST-Tat 1-101 (100 nM), recombinant human IFN-γ (1 µg/ml) or kept untreated. After 24 h MoDC were collected and analysed for both IDO-1 expression by western-blot and IDO activity (kynurenine production) following a 2 h incubation in HBSS completed with L-tryptophane (500 µM). (**D**) MoDC were treated with HIV-1 Tat protein (100 nM), in presence or absence of anti-TLR4 (HTA125, 5 µg/ml), anti-TLR2 (TL2.1, 5 µg/ml), isotype control antibodies (5 µg/ml), rTLR4/MD2 or rCD14 (5 µg/ml). 24 hrs after treatment cells were collected, lysed and analysed by SDS-PAGE and Western-Blot for IDO-1, and actin protein expression. (E) Recombinant GST-Tat 1-101 (10-100 nM) were incubated for 1 h at 37 °C in the presence or absence of recombinant TLR4/MD2 (5 µg/ml). Then the mixture was used to treat MoDC. After 24 h of treatment PD-L1 expression on MoDC cell surface was monitored by FACS and IL-10 production in the cell culture supernatant was quantified by ELISA. Histogram plot show FACS analysis of cell surface PD-L1 expression on MoDC either untreated or incubated with Tat 100 nM in presence or absence of rTLR4/MD2. The data represent means and SD for three independent experiments. Statistical significance was analyzed by one-way ANOVA follow by Bonferroni posttests and is marked as follows: *P < 0.05; **P < 0.01; ***P < 0.001; ns, nonsignificant. All bars were compared to those for untreated cells unless otherwise specified (indicated with a black line above the bars). (**F**) MoDC were treated with HIV-1 Tat protein (100 nM), in presence or absence of CLI-095 (1 µM) or DMSO (CLI-095 diluent). 24 hrs after treatment cells were collected, lysed and analysed by SDS-PAGE and Western-Blot for IDO-1, and actin protein expression.

We next tested the role of Tat-TLR4 interaction in the capacity of HIV-1 Tat protein to induce IDO-1. To this end, MoDC cells were pre-incubated with LPS-RS, a known specific antagonist of TLR4 pathway^45^, recombinant soluble TLR4 -MD2, as a decoy TLR4 receptor or anti-TLR4/MD2, as blockade antibodies. In these conditions, we clearly demonstrated a total inhibition of Tat-induced IDO-1 in the presence of LPS-RS (Fig. 3A and 3B, upper and lower panels) and recombinant TLR4/MD2 (Fig. 3C, upper and lower panels) and a strong inhibition in the presence of blocking anti-TLR4/MD2 monoclonal antibody, Mab HTA125, of the capacity of Tat to induce both IDO-1 protein expression and its related enzymatic activity (Fig. 3B, upper and lower panels). Interestingly, the IFN-γ induced IDO-1 was not significantly affected in the presence of any of these inhibitors (Fig. 3A–C, upper and lower panels). As control anti-TLR2, isotype control antibodies, or rCD14 fails to inhibit Tat-induced IDO-1 expression (Fig. 3D). These results are also in line with the capacity of LPS-RS and soluble TLR4/MD2 to inhibit IL-10 and PD-L1, two immunossuppressive factors also induced via the recruitment and activation of TLR4 pathway by HIV-1 Tat protein (Fig. 3E). More interestingly, we showed that the blockade of TLR4 signaling by CLI-095, a known inhibitor which acts by blocking the intracellular domain of TLR4, strongly inhibited Tat-induced IDO-1 (Fig. 3F). All these results suggest the essential role of TLR4 pathway in the activation of IDO-1 expression by HIV-1 Tat protein.

Because all TLR pathway lead to the activation of NF-κB, we thus evaluated the implication of NF-κB pathway in Tat-induced IDO-1.

### NF-κB activation, is essential for Tat-induced IDO-1

**Figure 4:**
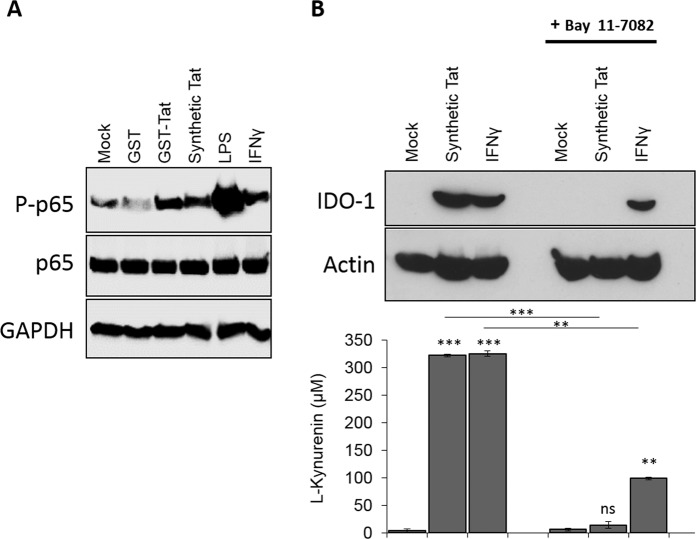
Tat induces IDO-1 in a NF-κB dependent manner in MoDC. (**A**) MoDC were incubated with recombinant GST (100 nM), GST-Tat (100 nM), synthetic Tat (500 nM), LPS (1 µg/ml) or IFN-γ (1 µg/ml) for 1 h . Then cells were lyzed and analyzed by immunoblots for Phospho-NF-κB p65 (Ser536) or total p65 protein. Detection of GAPDH was used as loading control. (**B**) MoDC were incubated with synthetic Tat (500 nM) or IFN-γ (1 µg/ml) in the presence or absence of NF-κB inhibitor Bay 11-7082 (5 µM) for 18 hrs. Then cells were collected and analyzed for IDO-1 protein expression by immunoblots and IDO activity by measuring L-Kynurenine following incubation for 2 h in HBSS completed with L-tryptophane (500 µM). The data represent means and SD for three independent experiments. Statistical significance was analyzed by one-way ANOVA follow by Bonferroni post tests and is marked as follows: *P < 0.05; **P < 0.01; ***P < 0.001; ns, nonsignificant. All bars were compared to mock-treated cells unless otherwise specified (indicated with a black line above the compared bars).

We tested the effect of Tat on NF-κB activation. To this end, human MoDC were left unstimulated or stimulated during 60 min with Tat protein or with the same concentration of GST as control. LPS and IFN-γ treatment were used as a positive control. After cell lysis, SDS-PAGE and protein blotting in the presence of phosphatase inhibitors, NF-κB activation was monitored by using specific antibodies against total p65 or phosphorylated p65, the Rel-A subunit of NF-κB. In these conditions, we clearly showed that HIV-1 recombinant as chemically synthetic Tat proteins induced the activation of NF-κB as demonstrated by the phosphorylation of p65 NF-κB subunit, while only a weak or no significant phosphorylation were observed in untreated or GST treated MoDC (Fig. 4A). Then, in order to test the role of NF-κB in Tat-induced IDO-1, MoDC were pretreated during one hour with non toxic amounts of Bay11-7082, a chemical known inhibitor of NF-κB, before treatment with Tat protein or IFN-γ. In these conditions, no IDO-1 protein expression was detected by western blot analysis in the protein cell lysates of Tat-treated MoDC in the presence of Bay11-7082 (Fig. 4B). In agreement with this latter result, no IDO-1 enzymatic activity was found as demonstrated by the absence of the kynurenine, in the enzymatic assay (Fig. 4B). However, when the expression of IDO-1 was induced by IFN-γ stimulation, only a partial inhibition of IDO-1 was observed, both at enzymatic and proteic levels (Fig. 4B) suggesting the recruitment of additional signaling pathways not sensitive to NF-κB inhibitor.

Together these experiments suggest the crucial role of Tat-TLR4/MD2 interaction and the downstream NF-κB pathway in the activation of the IDO-1 gene expression in human monocyte-derived dendritic cells. Taking into account this information, then, we asked if the expression of a functionally active TLR4/MD2 is sufficient to confer the capacity of HIV-1 Tat protein to induce the activation of IDO-1 gene expression.

### TLR4/MD2 expression on HeLa cell lines is not sufficient to drive IDO-1 expression by Tat

**Figure 5:**
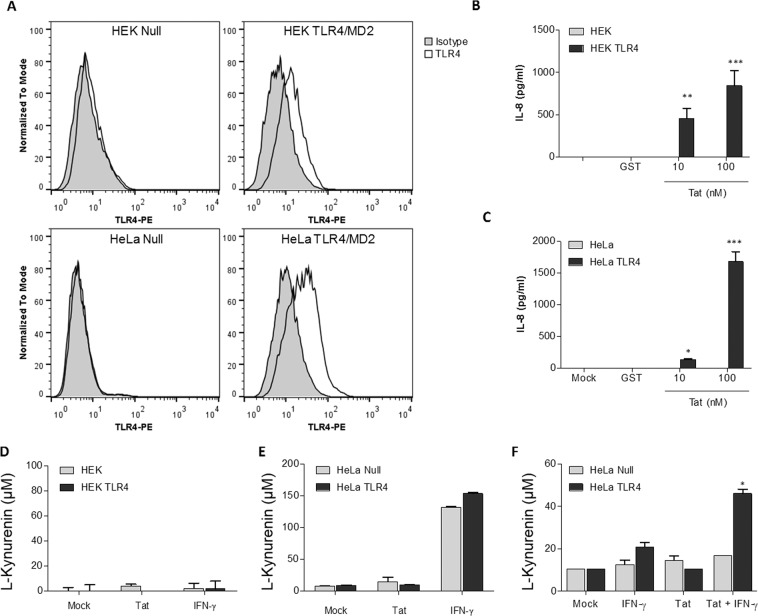
Activation of TLR4/MD2 pathway by Tat is necessary but not sufficient to induces the expression of IDO-1. (**A**) HeLa and HEK cell lines overexpressing TLR4/MD2-CD14 or transfected with empty plasmids (Null) were characterized for cell surface TLR4/MD2 expression by FACS. (**B**) HEK cell lines and (**C**) HeLa cell lines overexpressing TLR4/MD2 or not (Null) were incubated with recombinant GST-Tat (10, 100, nM), or GST (100 nM) as control. After 24 h of treatment IL-8 production in the cell culture supernatant was quantified by ELISA. (**D**) HEK cell lines and (**E**) HeLa cell lines overexpressing TLR4/MD2 or not (Null) were incubated with synthetic Tat (500 nM) or IFN-γ (1 µg/ml). After 24 h of treatment IDO activity was monitored by measuring L-Kynurenine following incubation for 4 h in HBSS completed with L-tryptophane (500 µM). (F) HeLa cell lines overexpressing TLR4/MD2 or not (Null) were incubated with synthetic Tat (500 nM), suboptimal amount of IFN-γ (100 ng/ml) or both Tat and IFN-γ (100 ng/ml). After 24 h of treatment IDO activity was monitored by measuring conversion of L-tryptophane in L-Kynurenine. The data represent means and SD for three independent experiments. Statistical significance was analyzed by one-way ANOVA follow by Bonferroni post tests and is marked as follows: *P < 0.05; **P < 0.01; ***P < 0.001; ns, nonsignificant. All bars were compared to those for mock-treated cells.

To evaluate if TLR4/MD2 pathway is sufficient to confer the capacity of Tat to induce IDO-1 production, we took advantage of HeLa and HEK cell lines, that are naturally defective for TLR4 and in which TLR4/MD2 pathway was restored by a stable transfection of TLR4, MD2 and CD14 genes. Firstly, we characterized by flow cytometry the expression of cell surface TLR4/MD2. As shown in Fig. 5A, a clear TLR4/MD2 labeling was detected in both TLR4/MD2 expressing HeLa and HEK cells, in comparison with the control HeLa and HEK cell lines (HeLa null, HEK null), thus demonstrating the cell surface expression of TLR4/MD2 (Fig. 5A). Furthermore, we next evaluated the biological functionality of TLR4 pathway in these cell lines, by testing the capacity of Tat protein to induce the production of IL-8 (CXCL-8) cytokine as described previously^44^. As depicted in Fig. 5B,C, HIV-1 Tat proteins are able to stimulate the production of IL-8 in a dose dependent manner in TLR4/MD2 expressing cells but not in null cell lines demonstrating the biological functionality of the TLR4 pathways. Then, we tested the capacity of IFN-γ to induce IDO enzymatic activity in both cell lines expressing or not TLR4. While no IDO activation was observed in HEK null or expressing TLR4/MD2-CD14 (Fig. 5D), similar functional IDO enzymatic activity was detected in HeLa null and HeLa TLR4/MD2 following IFN-γ treatment (Fig. 5E) demonstrating that HeLa cells, but not HEK cell, are able to express IDO-1 in response to IFN- γ stimulation (Fig. 5D,E). However, despite cell surface expression of TLR4/MD2 and the biological functionality of TLR4 pathway, Tat protein is not able to drive IDO activity neither in HeLa cells nor in HEK cell lines expressing TLR4/MD2-CD14 (Fig. 5D,E). These results demonstrate that in addition to TLR4/MD2-CD14, additional pathways are required to confer the capacity of Tat to induce IDO-1. Thus, we tested the effect of IFN-γ priming of HeLa cells with suboptimal amount of IFN-γ that are not sufficient to trigger IDO enzymatic activity in HeLa cells (Fig. 5F) in combination with Tat treatment. The results depicted in Fig. 5F show that suboptimal amount of IFN-γ restored the capacity of Tat to induce the expression of IDO enzymatic activity in HeLa-TLR4/MD2-CD14 cell line but not in HeLa null cell line (Fig. 5F). These results demonstrate that TLR4/MD2-CD14 is necessary but not sufficient to confer the capacity of Tat to induce IDO-1 at least in HeLa cells and suggest the involvement of additional pathways activated by Tat protein which are unlocked following IFN-γ treatment.

## Discussion

In the present study we demonstrated that HIV-1 Tat protein induced the production of active IDO-1 protein in human MoDC by recruiting TLR4 pathway. In addition to Tat protein^36–38^, other HIV-1 viral factors have also been reported for their capacity to stimulate IDO-1 expression or activity, including Nef protein through an unknown mechanism^46^ and gp120^47^ following direct binding to CD4 or viral ssRNA via TLR7 pathway^47,48^.

By its capacity to catabolize L-tryptophan, IDO-1 has been initially described for its anti-viral and anti-bacterial activities which are essentially mediated by a mechanism involving tryptophan starvation. However, in the course of HIV-1 infection, the activity of IDO, evaluated by measuring the concentration of kynurenin in the plasma of HIV-1 infected patients, seems to increase with the progression of the disease to the AIDS stage^49^ suggesting a deleterious effect of IDO expression. This IDO activity has been associated with several pathological effects including immunological disorders, inefficient immune responses, T cell exhaustion and neurological dysfunction^50^. Additional studies reported a pleiotropic involvement of IDO in a variety of pathophysiological processes such as autoimmune diseases, allergies and different types of cancers essentially by establishing a tolerogenic state^39,51^.

Despite its pathological effect following dysregulated expression in several settings it was reported that IDO-1 is constitutively expressed by extravillous trophoblasts and plays a major role in immune tolerance at the foeto-maternal interface to prevent fetuses rejection^52,53^. A crucial beneficial role of IDO-1 is also involved in the tolerance of allograft transplantation.

Because Tat protein is able to stimulate the expression of a large variety of genes including IFN-γ, a cytokine known for its potent capacity to stimulate the induction of IDO-1, we evaluated if the induction of IDO-1 by Tat is mediated by a direct action of Tat or by an indirect mechanism involving Tat induced factors. To this end we demonstrated that the induction of IDO-1 by Tat required a direct interaction between Tat and MoDC and exclude a mechanism involving soluble factors secondary induced by Tat, such as IFN-γ for at least two reasons: (i) time courses experiments showed that Tat induced IDO-1 production appeared since 12 hours post treatment, while IFN-γ induction by Tat requires 24 h^36^, (ii) the induction of IDO-1 by IFN-γ, but not by Tat protein, can be totally inhibited in the presence of inhibitor of IFN-γ pathway^36^. In agreement with a direct action of Tat, we demonstrated that the induction of IDO-1 is independent of other Tat induced secondary factors. Altogether these arguments demonstrated that HIV-1 Tat protein induced IDO-1 production by a mechanism involving a direct interaction between Tat and MoDC.

In addition to IDO-1, we have, previously, shown that HIV-1 Tat protein also, stimulated, in human monocytes and dendritic cells, the production of IL-10, a highly immunosuppressive cytokine^28^ and PD-L1, an essential immune checkpoint^35^. These two factors were reported to be involved in the immune disorders observed in HIV-1 infected patients^54–56^. Exploration of the molecular mechanism and signaling pathway recruited by Tat to stimulate IL-10 and PD-L1, showed that Tat protein recruits TLR4 signalling pathway and activates both MyD88 and Trif pathways^14,57^. However we showed that Tat induced IL-10 and PD-L1 through an indirect mechanism involving TNF-α, as demonstrated by the capacity of anti-TNF-α to inhibit partially, Tat-induced IL-10^17^ or totally, Tat-induced PD-L1 up-regulation on MoDC^35^.

Although Tat induced the production of IDO-1 in a direct mechanism, the mode of action of Tat can be mediated either at the intracellular or at the cell surface level. To answer that question, we showed that the N-terminal fragment Tat 1-45, deleted from the basic domain region which is essential for Tat internalization, retained its capacity to induce IDO-1 production^36^. Thus, our data argued that Tat protein, mediated its effect by a mechanism acting at the cell membrane level. Furthermore, this induction of IDO-1 by Tat seems to be dependent, at least, on the secondary structure of Tat protein, since the stimulation with oxidized Tat protein abolished totally its capacity to stimulate IDO-1 production. This latter observation seems also to be related to the need of a relatively high concentration (500 nM) of synthetic Tat protein, a preparation which seems to contain only a small fraction of native-like Tat protein.

Among the variety of cell membrane receptors reported for Tat protein, including, CD26 receptor^30^, L type calcium channel^31^, integrins αvβ5 and α5β5 FLK1/KDR receptor^32^ or TLR4^14^, we hypothesized the implication of TLR4 pathway in the induction of IDO-1 by Tat. In favor of that hypothesis we showed that in addition to the capacity of Tat protein to interact with TLR4/MD2 complex in a solid-phase binding assay, blockade of TLR4 pathway with either anti-TLR4/MD2 antibodies, TLR4 antagonist LPS-RS or soluble recombinant TLR4/MD2 proteins inhibited partially or totally the induction of IDO-1 protein expression by Tat as well as IDO-1 activity. It is interesting to note that IFN-γ-induced IDO-1 was not significantly affected in the presence of LPS-RS, anti-TLR4/MD2 antibodies, or soluble recombinant TLR4/MD2 proteins. This latter result represents a second argument in favor of a specific and selective mechanism of Tat-induced IDO-1 different from that previously described for IFN-γ.

Because TLR4 pathway is necessary to confer the capacity of Tat to induce IDO-1 protein expression and activity in MoDC, we then aimed to determine if it is sufficient. To investigate that question, we used HEK and HeLa cell lines that are naturally defectives for TLR4 pathway, and then, we evaluated if trans-expression of a functional TLR4 pathway in these two cell lines, could be enough to restore the capacity of Tat to stimulate IDO-1 expression. We first characterized that in both TLR4, MD2 and CD14-transfected cell lines, TLR4 is expressed at the cell membrane level, by flow cytometry analysis. In addition TLR4 pathway is functional as demonstrated by the capacity of Tat protein to stimulate the expression of IL-8 in a TLR4-dependent manner. Despite this functional expression of TLR4 pathway, Tat protein remained unable to stimulate the production of IDO-1, suggesting that the expression of TLR4 pathway is not sufficient to restore the capacity of Tat to induce the production of IDO-1 in HeLa and HEK cell lines. However, IFN-γ failed to trigger IDO-1 expression in HEK cells suggesting that HEK are probably defective for IDO-1. By contrast, HeLa cell were able to produce enzymatically active IDO-1 following stimulation by IFN-γ in a TLR4/MD2 independent manner. This result represents an additional characterization in favor of the hypothesis that Tat and IFN-γ induced IDO-1 through different mechanisms.

At the transcriptional level, the regulation of IDO-1 gene expression seems to be dependent on the activation of several transcription factors as shown by the presence of specific binding sites in the promoter of IDO-1 including, ISRE (interferon sequence response element), GAS (gamma activated sequence) and NF-κB binding sites^58^. Also it was reported that IDO-1 gene expression can be modulated positively by Forkhead box 3 (FOXO3) and IRF8, while it is repressed by DNAX-activation protein 12 (DAP12)^59^. IDO-1 is also regulated negatively at post-translational level by SOCS proteins which binds to IDO-1 and promotes its ubiquitinylation and degradation via the proteasome pathway^60^.

In the present study, we evaluated the implication of transcription factor NF-κB in the control of IDO-1 expression following stimulation by Tat. To this aim, we showed on the one hand that HIV-1 Tat protein activated NF-κB in MoDC, as shown by the detection of phosphorylated p65 in Tat-treated MoDC. On the other hand, we showed that preincubation of MoDC with NF-κB inhibitor Bay 11-7082, abrogated the capacity of Tat to induce IDO-1 expression in MoDC. However, it is interesting to note that despite a partial reduction, IFN-γ is still able to induce IDO-1 protein expression and activity in the presence of this inhibitor. This difference suggests the implication of redundant involvement of other transcription factors activated by IFN-γ that are not sensible to the inhibition by the used chemical NF-κB inhibitor.

In conclusion, the present study underlines the importance of TLR4 signalling pathway in the capacity of HIV-1 Tat protein to stimulate the production of IDO-1 in human monocytes derived dendritic cells in a NF-κB dependent manner. Our study also clearly demonstrated that the presence of a functional TLR4 pathway seems not sufficient for Tat to induce the production of IDO-1 in HeLa or HEK cell lines suggesting the implication of additional stimuli which remain to be identified.

## Supplementary information

Supplementary Figure 1.

## Data Availability

The datasets generated during and/or analysed during the current study are available from the corresponding author on reasonable request.
