# Supplementary material for: HIV-1 Tat – TLR4/MD2 interaction drives the expression of IDO-1 in monocytes derived dendritic cells through NF-κB dependent pathway
Source: Sci Rep. 2020 May 18;10:8177. doi: 10.1038/s41598-020-64847-y (PMC7235218; doi:10.1038/s41598-020-64847-y)

HIV-1 Tat – TLR4/MD2 interaction drives the expression of IDO-1 in monocytes derived dendritic cells through NF-κB dependent pathway

Elmostafa Bahraoui[1,2,3*], Manutea Serrero[1,2,3], Rémi Planès[1,2,3*]

[1] INSERM, U1043, CPTP, CHU purpan, Toulouse, France

[2] CNRS, U5282 CPTP, CHU purpan, Toulouse, France

[3] Université Paul Sabatier, CPTP, CHU purpan, Toulouse, France

[*] Corresponding authors

remi.planes@ipbs.fr / elmostafa.bahraoui@univ-tlse3.fr

Supplementary material

We stated that the immunoblot presented in supplementary material correspond to the full length Immunoblot film/images without any modification or typesetting.

Figure S1

A) Original Immunoblot film related to Figure 1A

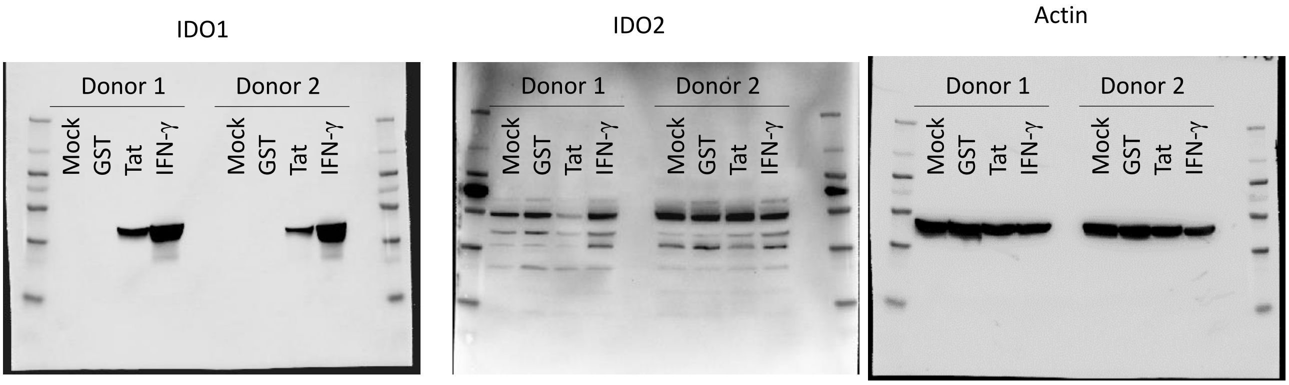

B) Original Immunoblot film related to Figure 1B

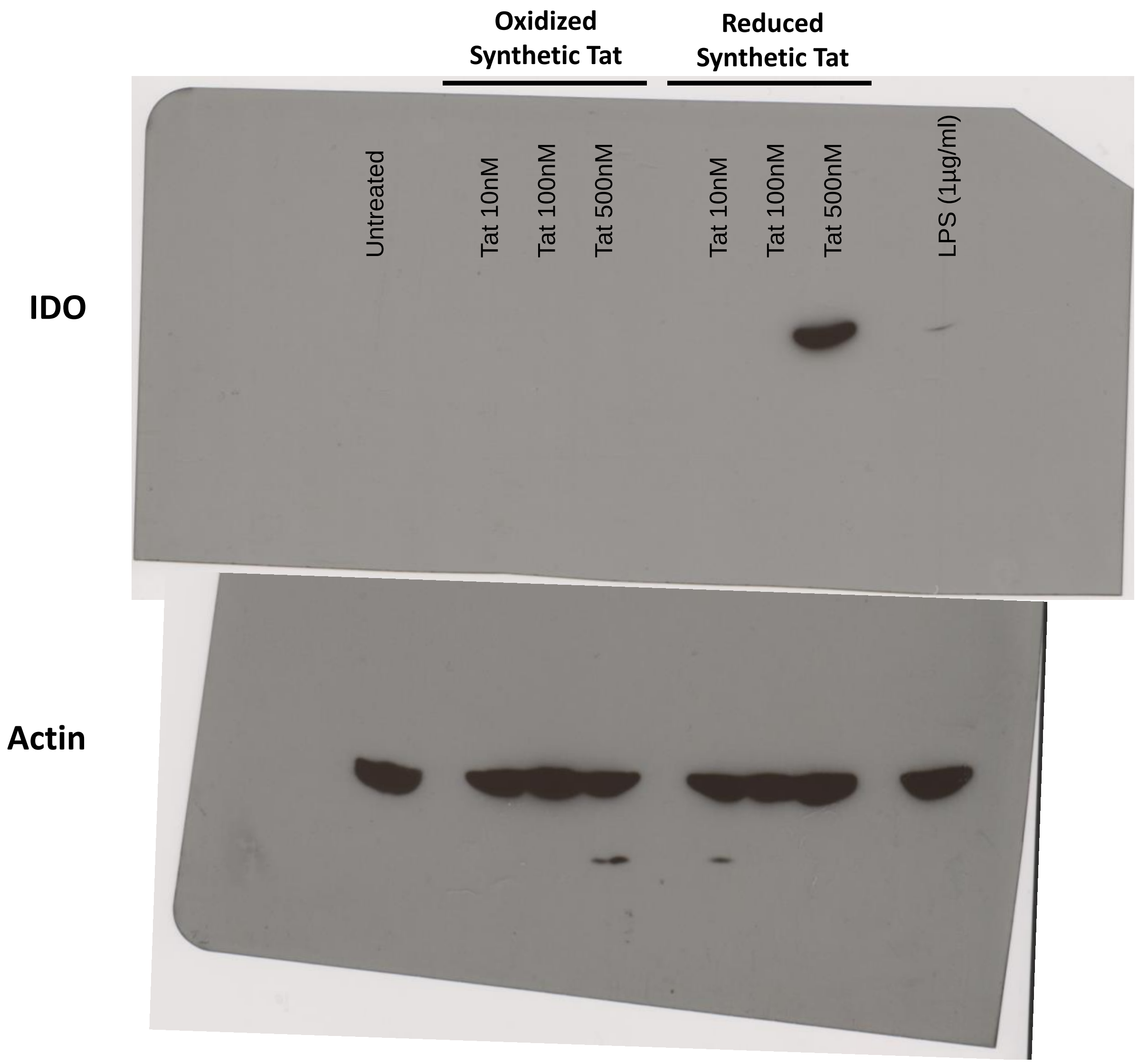

C) Original Immunoblot film related to Figure 3A

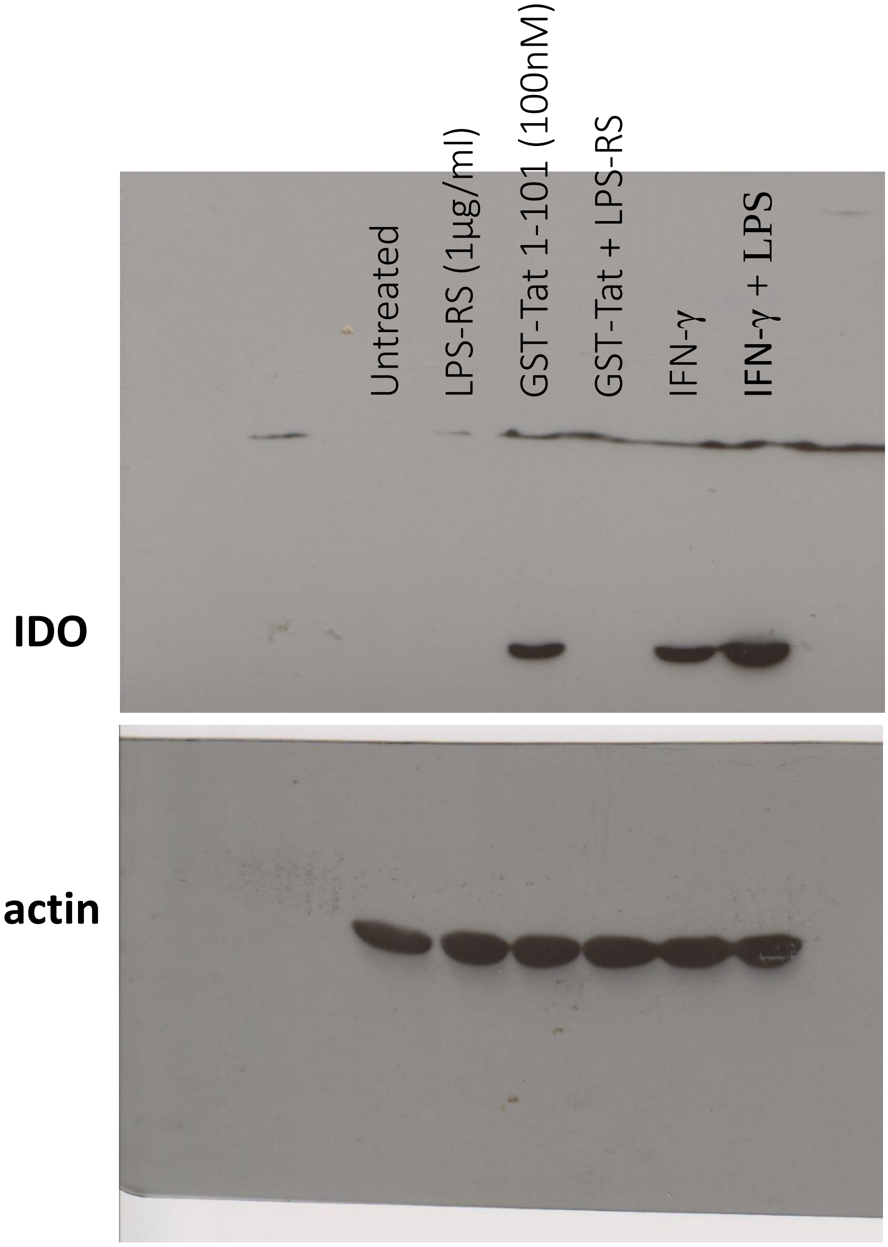

D) Original Immunoblot film related to Figure 3B

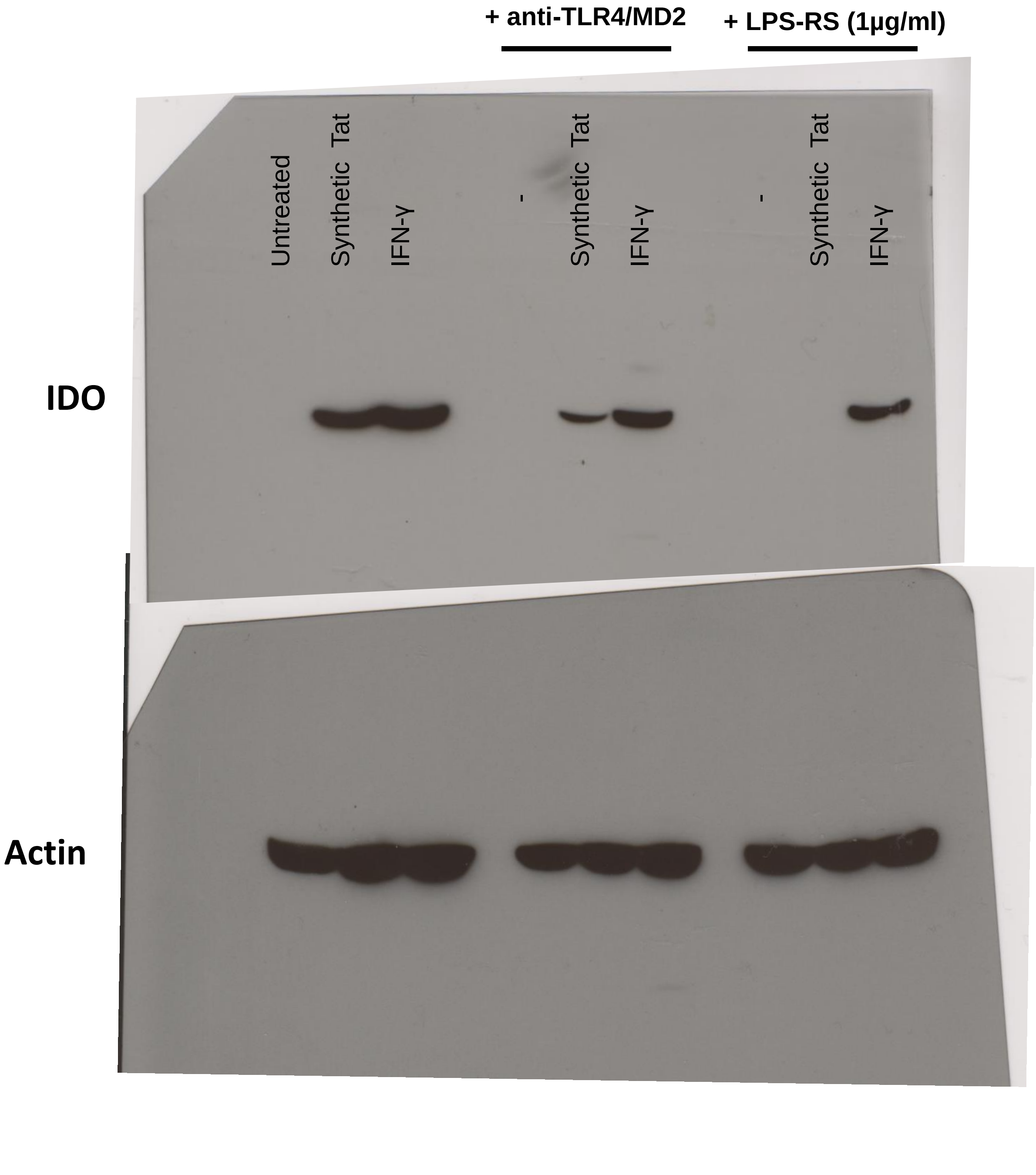

E) Original Immunoblot film related to Figure 3C

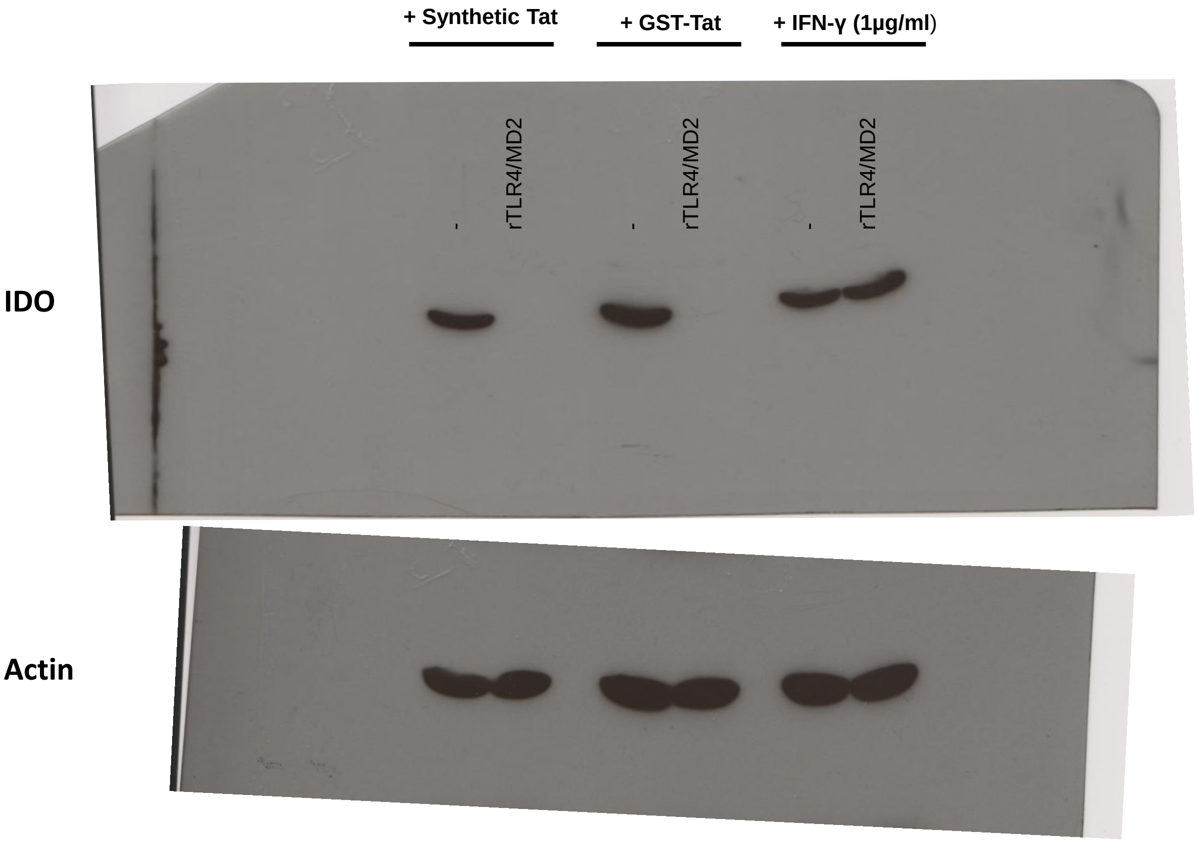

F) Original Immunoblot film related to Figure 3D

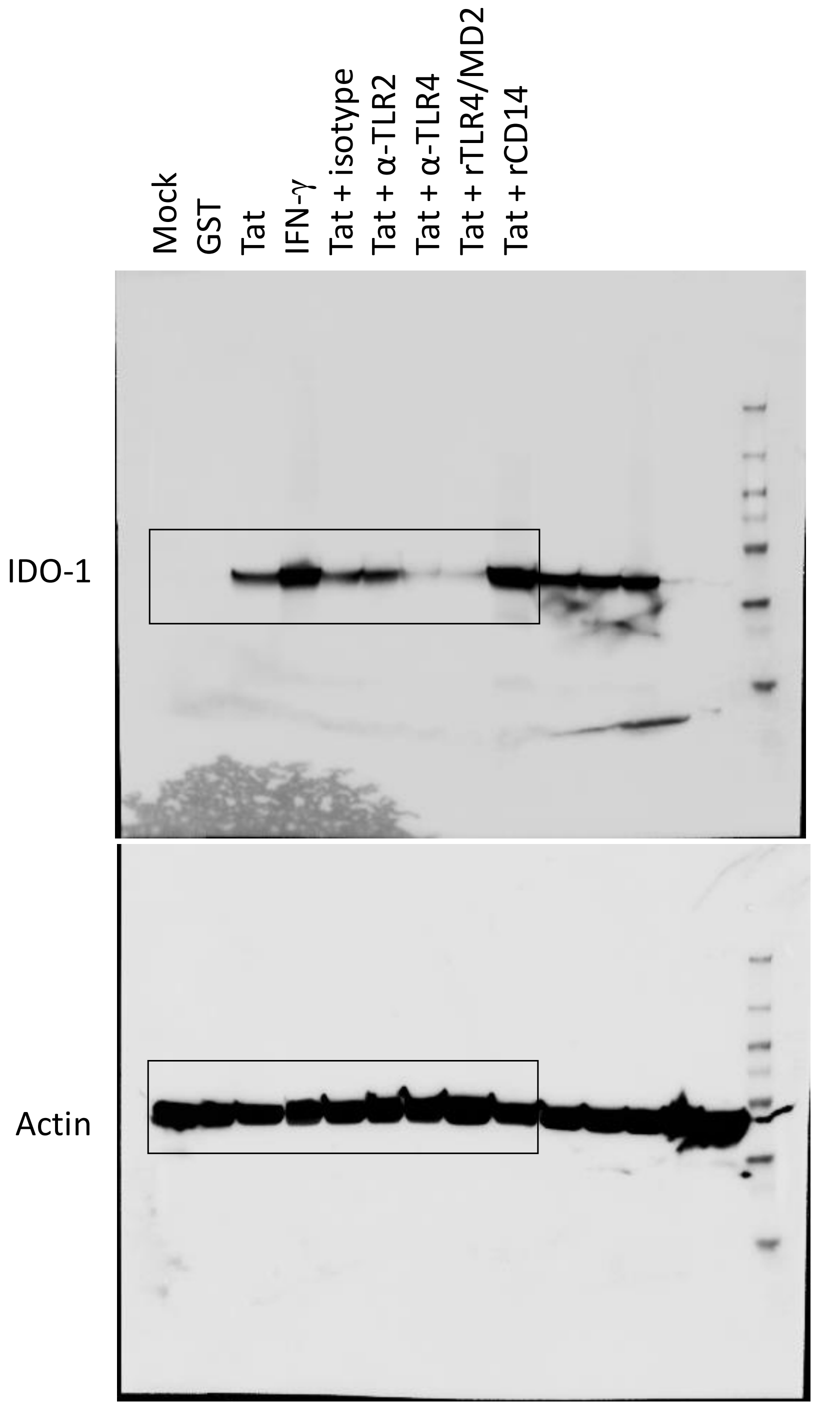

G) Original Immunoblot film related to Figure 3F

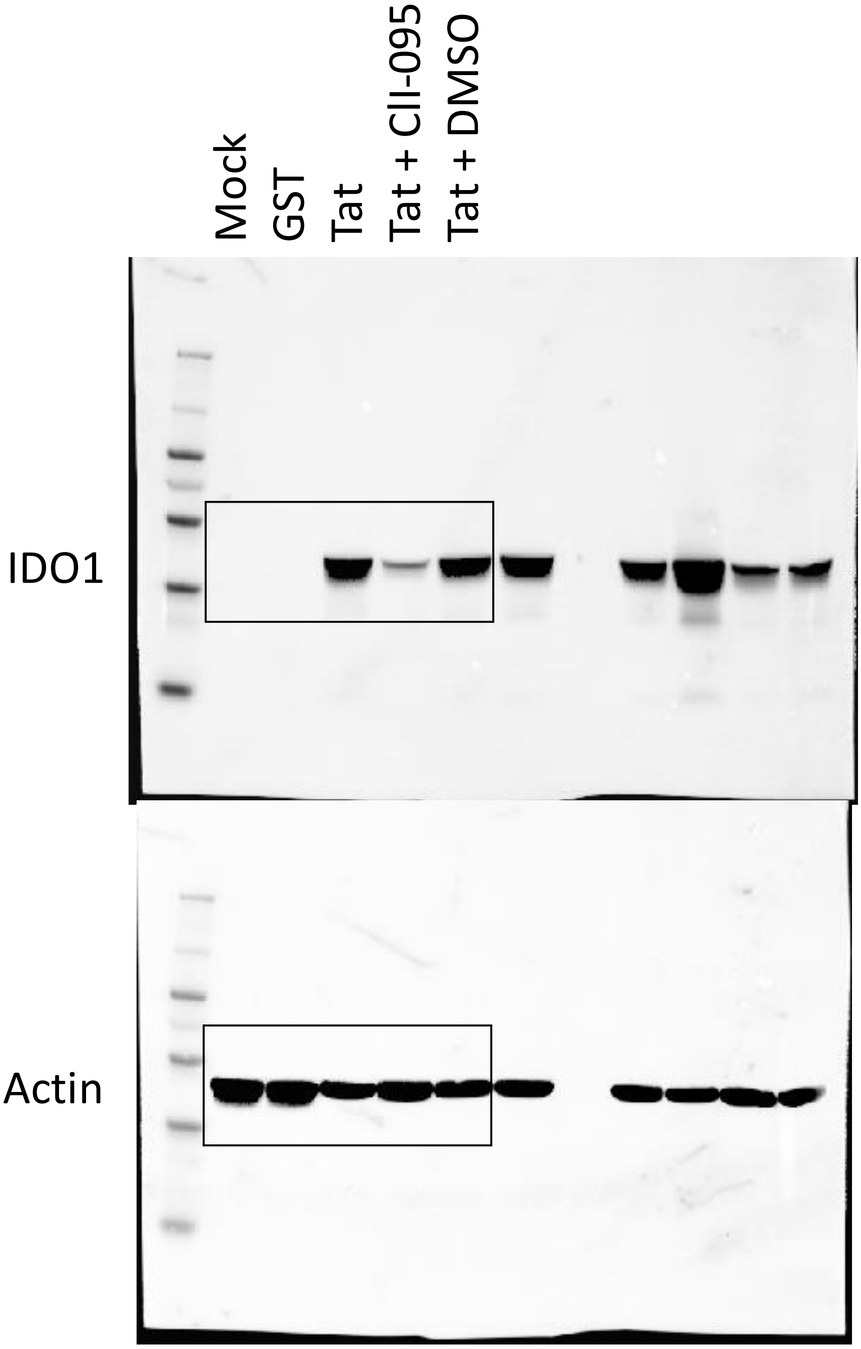

H) Original Immunoblot images related to Figure 4A

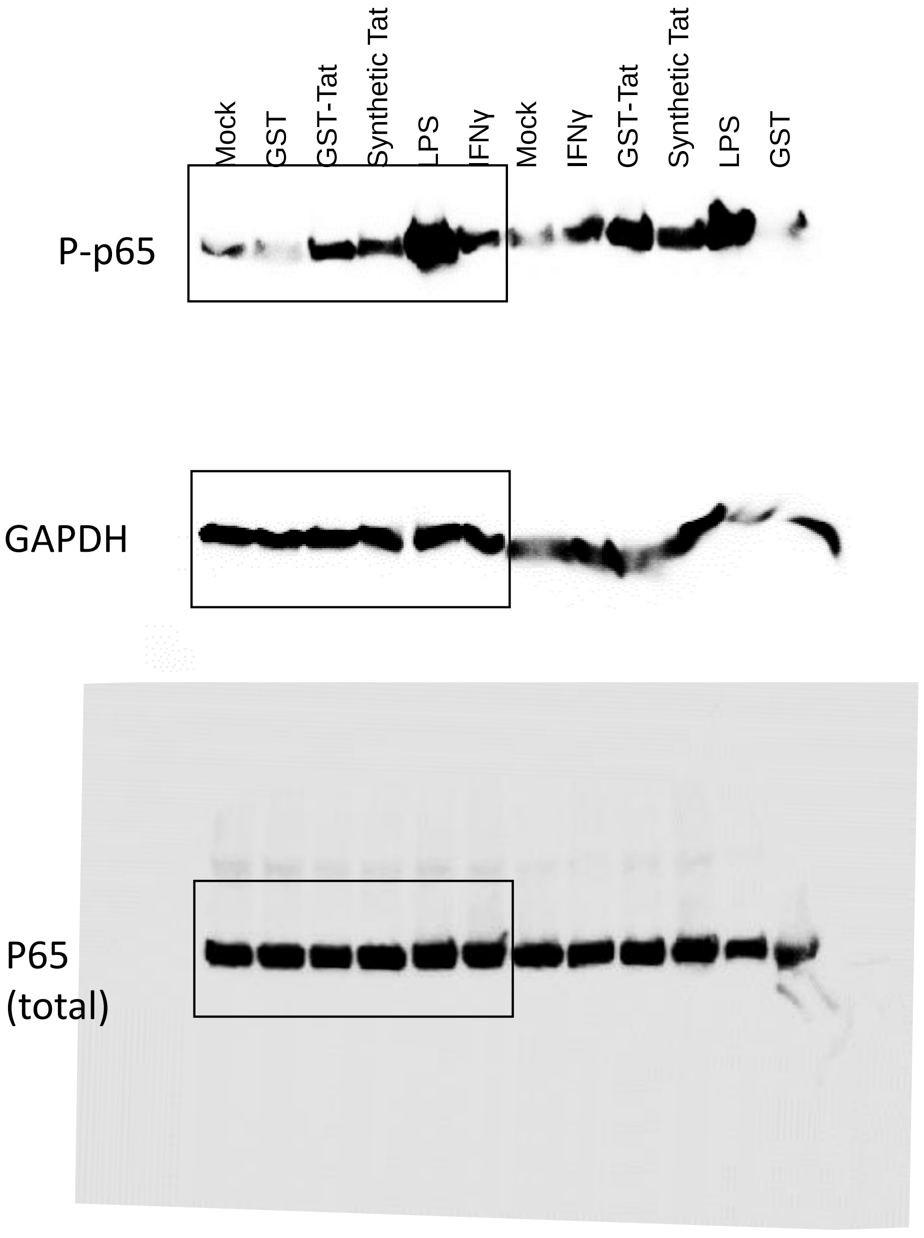

I) Original Immunoblot film related to Figure 4B

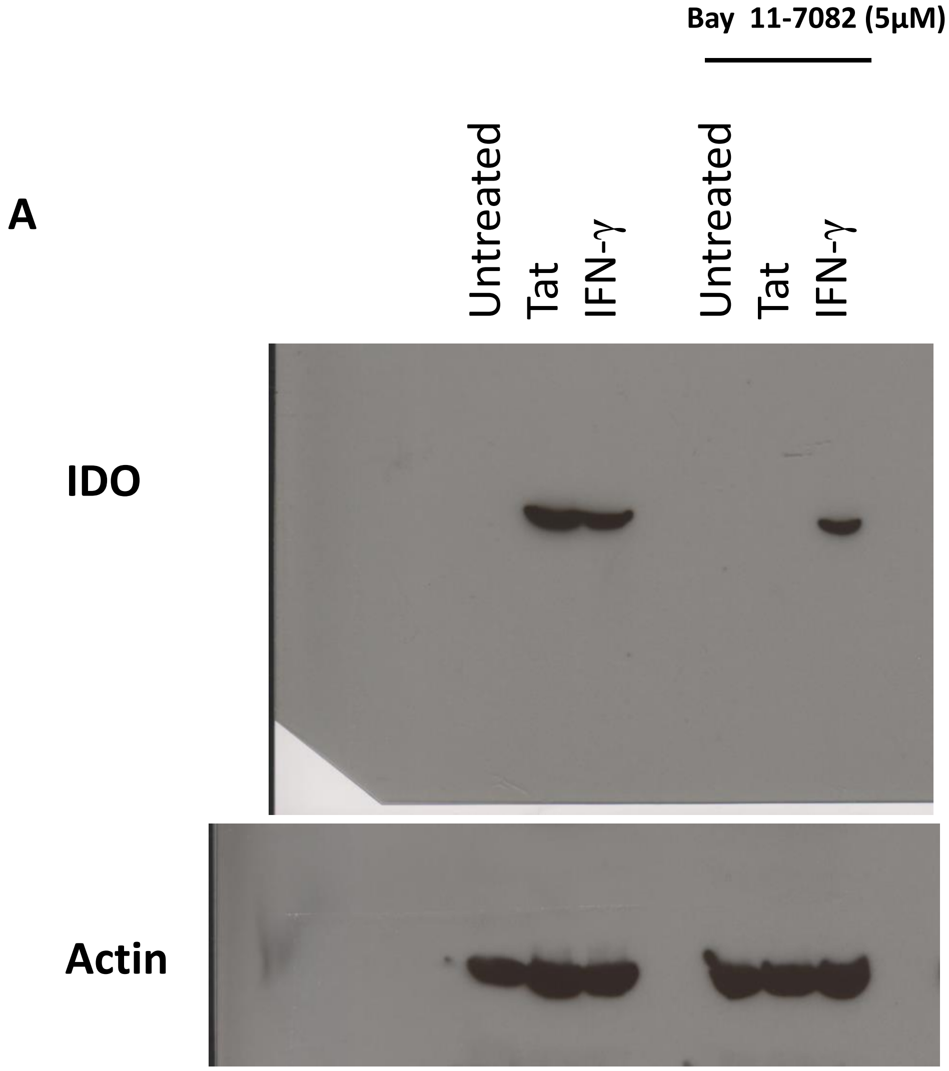

Supplement: Supplementary file 1 — Supplementary Figure 1. [file 41598_2020_64847_MOESM1_ESM.pdf]
